# The COVID-19 vaccination decision-making preferences of elderly people: a discrete choice experiment

**DOI:** 10.1038/s41598-023-32471-1

**Published:** 2023-03-31

**Authors:** Yuhan Chen, Jimeng Wang, Meixi Yi, Hongteng Xu, Hailun Liang

**Affiliations:** 1 School of Public Administration and Policy, Renmin University of China, Beijing, 100872 China; 2 Gaoling School of Artificial Intelligence, Renmin University of China, Beijing, China; 3 School of Philosophy, Renmin University of China, Beijing, China

**Keywords:** Health policy, Public health

## Abstract

COVID-19 is a continuing threat to global public health security. For elderly people, timely and effective vaccination reduces infection rates in this group and safeguards their health. This paper adopted an offline Discrete Choice Experiment (DCE) to research the preference for COVID-19 vaccination amongst Chinese adults aged 50 years and above. Through multinomial logistic regression analysis, our DCE leverages five attributes—the risk of adverse reactions, protective duration, injection doses, injection period, and effectiveness—each of which is split into three to four levels. The risk of adverse reaction and the protective duration were demonstrated to be determinants of vaccination preference. Moreover, it was found that socio demographic factors like region, self-health assessment and the number of vaccinated household members can strengthen or weaken the effects of vaccine attributes. In conclusion, the preferences of the elderly population should be considered when developing COVID-19 vaccination programs for this population in China. Accordingly, the results may provide useful information for policymakers to develop tailored, effectively vaccination strategies.

## Introduction

COVID-19 is a novel coronavirus strain that can cause symptoms ranging from mild to severe illness. The virus is characterised by its high infectivity and concealment^1^. The COVID-19 pandemic has led to a large number of deaths and incalculable economic losses globally. Developing effective vaccines to prevent further casualties and property losses in a timely manner, is the only feasible means to alleviate the ongoing public health crisis^2^. The high degree of danger associated with the spread of COVID-19 renders vaccine development a priority. Vaccination plays a significant role in preventing serious diseases whilst also reducing hospitalization and mortality rates^3^. According to US CDC statistics, compared with unvaccinated people, vaccination reduces the risk of novel coronavirus infection by 5 times, the risk of hospitalization by a factor of at least 10, and the risk of death by 10 times^4^. To date, several different types of COVID-19 vaccines like protein subunit vaccine, RNA based vaccine and etc., have been developed worldwide and some have passed all the three clinical phases; but due to the short time frame for their development and clinical trials, the properties including effectiveness, safety and adverse reaction of vaccines have yet to be revealed, and therefore people’s willingness and acceptance of vaccination was not high. Given the contradiction between the trend toward widespread adoption and people’s hesitation to vaccinate, and in order to gain a more thorough insight into the reasons for non-compliance to vaccination, it is of vital significance to investigate and analyze patients’ inoculation decision-making^5^. Also, in terms of the target groups, older people are often not a priority population for vaccine trials due to many susceptible factors^6^. In addition, COVID-19 disease-related deaths are higher in older populations due to their weakened immune systems and higher rates of chronic disease. Therefore, it’s of great significance for the elderly population to get vaccination and the vaccine promotion among this group needs to be more carefully. The purpose of this study was to use a Discrete Choice Experiment (DCE) to explore the preferences of Chinese people aged 50 years and over relating to COVID-19 vaccination. It is hoped that the findings presented here will help inform health policy and improve vaccination coverage and performance^7^. Given the present circumstances, it is of great significance to measure the preference of vaccination decision-making more accurately, so as to improve patients’ vaccine acceptance.

The health belief model (HBM) is a theory that uses a psychosocial approach to explain and predict health-related behaviours^8,9^. It assumes that an individual’s likelihood of performing a health behaviour is determined by four main dimensions: perceived susceptibility, perceived severity, perceived benefits and perceived barriers. A person's perceptions of action could shape action expectations, which further influence the likelihood of taking action, sustaining action and forming habits^10^. HBM is used extensively in healthcare and health-related fields such as tuberculosis screening, cancer screening, dental diagnosis, etc^10–12^. More recently, research on COVID-19-related themes has increasingly applied HBM^13^, and this model has been widely used to explore the factors and mechanisms that affect vaccination behaviour^14^. Therefore, it is applied in this study to investigate into the specific factors of attitudes’ impact on behavioral intentions and the implementation process.

There are several models commonly used to predict patient preferences. For instance, multi-criteria decision analysis (MCDA) is a general method, which breaks down decisions into their component parts and systematically deals with them to support decision-making^15^. Another widely used method is the Analytic hierarchy process (AHP), which provides the order of the problem or objective and is used to guide decision-making in a formal hierarchical structure^16^. The model chosen in this study, the Discrete Choice ExperimFent (DCE), is an MCDA technique, which can be relied upon to predict patient preferences and improve vaccine acceptance by matching patient needs. The DCE is a survey widely used in patient-oriented research. Specifically, DCE is well-suited to determining patients’ preferences for different hypothetical treatment options by quantifying their preferences for various treatment decision attributes^17^. In doing so, DCE can estimate the preferences of complex objects by building assumptive scenarios, which allows for better manipulation and control of the selection situation^18^. To date, few empirical studies have been carried out using the DCE method to study COVID-19 vaccination preferences among elderly people. Therefore, utilizing the DCE to estimate the preferences of aging persons for the COVID-19 vaccine is both feasible and promising.

The present study aims to investigate the preferences of people aged 50 and above for COVID-19 vaccination using the Discrete Choice Experiment (DCE) method, providing a meaningful reference for policymakers and vaccine follow-up research based on the health belief model^19^. At the practice level, China encourages vaccination but has not yet completed nationwide. It is of great significance to measure the preference of vaccination decision-making more accurately so that the patient’s acceptance and willingness of the vaccine can get improved and equally revealing for the promotion of other vaccines. And these findings will help inform health policy and improve vaccination coverage and performance^7^. At the theoretical level, this study makes efforts in expanding the dimensions of research on COVID-19 vaccination and transmission further enriches the application areas of HBM theory.

## Results

### Respondents’ characteristics

A total of 298 questionnaire responses were received, of which 293 were valid (with a response rate of 98.33%). The demographic characteristics of participants are detailed in Table 1. Among the 293 subjects, 91 were men (31.1%) and 202 were women (68.9%), whilst over half were aged between 60 and 70 years old. Most were urban citizens (94.9%) and were retired (94.9%). Meanwhile, 134 individuals (45.7%) had an educational level of high school and technical secondary school, and 36.2% had bachelor’s diplomas or above, whilst the remainder had completed a middle school education or lower. About half of the respondents (51.9%) had an annual income of less than 50,000 Chinese Yuan and the majority of the participants (87.7%) had an annual income of less than 100,000 Chinese Yuan.

**Table 1:** Demographic characteristics of participants in this study.

Variable (n, %)	n (%) (N = 293)	Willing to be Vaccinated Against COVID-19	
		Yes (n = 200)	No (n = 93)
Gender			
Male	91 (31.1)	66 (33)	25 (26.9)
Female	202 (68.9)	134 (67)	68 (73.1)
Age			
51–55	14 (4.8)	11 (5.5)	3 (3.2)
56–60	47 (16.0)	30 (15.0)	17 (18.3)
61–65	101 (34.5)	74 (37.0)	27 (29.0)
66–70	68 (23.2)	48 (24.0)	20 (21.5)
71–75	24 (8.2)	15 (7.5)	9 (9.7)
76–80	15 (5.1)	6 (3.0)	9 (9.7)
81–85	11 (3.8)	8 (4.0)	3 (3.2)
86–90	9 (3.1)	6 (3.0)	3 (3.2)
91–95	4 (1.4)	2 (1.0)	2 (2.2)
Annual income			
< 50,000 yuan	152 (51.9)	96 (48)	56 (60.2)
50,000–100,000 yuan	105 (35.8)	83 (41.5)	22 (23.7)
> 100,000 yuan	36 (12.3)	21 (10.5)	15 (16.1)
Main source of income			
Savings income	1 (0.3)	1 (0.5)	0 (0.0)
Wage income	27 (9.2)	17 (8.5)	10 (10.8)
Pension fund	252 (86.0)	176 (88.0)	76 (81.7)
Other income	7 (2.4)	3 (1.5)	4 (4.3)
Children’s support	6 (2.0)	3 (1.5)	3 (3.2)
Region			
City	278 (94.9)	189 (94.5)	89 (95.7)
Rural	15 (5.1)	11 (5.5)	4 (4.3)
Educational level			
Below junior high school	53 (18.1)	32 (16.0)	21 (22.6)
High school and technical secondary school	134 (45.7)	88 (44.0)	46 (49.5)
Bachelor and college	96 (32.8)	72 (36.0)	24 (25.8)
Graduate and above	10 (3.4)	8 (4.0)	2 (3.2)
Employment status			
Work	15 (5.1)	11 (5.5)	4 (4.3)
Retirement	278 (94.9)	189 (94.5)	89 (95.7)
Underlying history of chronic illness			
Yes	141 (48.1)	89 (44.5)	52 (55.9)
No	152 (51.9)	111 (55.5)	41 (44.1)
Evaluation of their own health status			
Very poor	4 (1.4)	3 (1.5)	1 (1.1)
Poor	15 (5.1)	5 (2.5)	10 (10.8)
Fair	142 (48.5)	96 (48.0)	46 (49.4)
Good	105 (35.8)	74 (37.0)	31 (33.3)
Very good	27 (9.2)	22 (11.0)	5 (5.4)
Approximate distance from place of residence to a fixed place for medical treatment			
Within 1 km	60 (20.5)	43 (21.5)	17 (18.3)
1–3 km	87 (29.7)	63 (31.5)	24 (25.8)
3–7 km	51 (17.4)	39 (19.5)	12 (12.9)
Over 7 km	25 (8.5)	14 (7.0)	11 (11.8)
No	70 (23.9)	41 (20.5)	29 (31.2)
Focus on the research and development progress of the COVID-19 vaccine			
Don’t focus on	12 (4.1)	2 (1.0)	10 (10.8)
A little attention	41 (14.0)	17 (8.5)	24 (25.8)
More attention	99 (33.8)	65 (32.5)	34 (36.5)
Pay close attention to	138 (47.1)	114 (57.0)	24 (25.8)
Don’t know	3 (1.0)	2 (1.0)	1 (1.1)
The number of family members who have been vaccinated			
0 people	116 (39.6)	62 (31.0)	54 (58.1)
1–2 people	138 (47.1)	104 (52.0)	34 (36.6)
3–4 people	31 (10.6)	28 (14.0)	3 (3.2)
More than 4 people	8 (2.7)	6 (3.0)	2 (2.2)
Willing to be vaccinated against COVID-19			
Yes	200 (68.3)	200	0
No	93 (31.7)	0	93
Participation in the health care			
No	25 (6.6)	19 (7.4)	6 (5.0)
Basic medical insurance for urban workers	170	127	43
Basic medical insurance for non-working urban residents	66	39	27
The new type of rural cooperative medical care	11	6	5
Free medical care	67	45	22
Basic medical insurance for urban and rural residents	12	6	6
Other	26	15	11

In the respect of vaccine-related patient characteristics, 48.5% of the respondents’ self-health assessment were fair and 35.8% thought themselves in good condition. 141 respondents had a history of chronic disease. 76.1% reported that they had fixed sites for medical treatments. Most respondents paid high or relatively high attention to the research and development of COVID-19 vaccines and 68.3% were willing to undergo vaccination. Meanwhile, 60.4% of the sample indicated their families have been vaccinated. Further, 98.6% received some form of medical insurance, of which the number of basic medical care for urban workers (52.2%) and public health care (20.1%) were relatively higher.

Furthermore, we divided the respondents into two groups according to their willingness to get vaccinated, and relatively significant differences in their demographic characteristics were in the following areas. Firstly, a higher proportion of men than women were willing to be vaccinated. Secondly, among those who were unwilling to get vaccinated, a higher proportion had an annual income of less than 50,000 Chinese Yuan and a lower level of education, and a higher proportion had a chronic underlying illness or a poorer self-assessed health status than those who were willing to be vaccinated.

### Differences in attributes and levels

As the number of vaccination injection doses and the interval are typically jointly determined, we combined these two attributes to create a new variable for analysis, vaccine injection time. Table 2 shows that all the attributes were significantly related to respondents’ vaccination preference, while the levels that protective duration was 24 months was not statistically significant. From this, it can be suggested that these attributes impact elderly residents’ decisions to get a COVID-19 vaccination. Moreover, it is apparent that the risk of adverse reaction was the most critical factor, which was negatively significant for vaccination intention, indicating that the safety of vaccines was of the greatest concern to seniors, especially whether there were serious risks to their health. The next most important attribute was the protective duration, with the level of 18 months being significantly associated with people's willingness to receive a vaccination (odds ratio = 2.766, p < 0.001). Vaccine injection time was a hazard factor and had moderately strong association with willingness to vaccinate. The variable of 80% effectiveness was negatively related to vaccination preference (odds ratio = 0.447, p < 0.001), which required further investigation.

**Table 2:** Regression results of vaccination preferences on different attributes and levels.

	Coefficient(SE)	Standardized Coefficient	P value	Exp (B)	95% CI for Exp (B)	
					Lower	Upper
Risk of adverse reaction (No side effects^a^)			< 0.001***			
Local or systemic adverse reactions	− 1.217(0.249)***	− 0.419***	< 0.001***	0.296	0.182	0.483
Severe adverse reactions	− 1.229(0.175)***	− 0.423***	< 0.001***	0.292	0.208	0.412
Protective duration (6 or 12 months)			< 0.001***			
18 months	1.017(0.163)***	0.354***	< 0.001***	2.766	2.009	3.809
24 months	− 0.315(0.193)	− 0.11	0.103	0.730	0.500	1.066
Vaccine injection time (1 dose)			0.001***			
2 doses with the interval of 14/28 days	0.491(0.169)**	0.219**	0.004**	1.634	1.174	2.274
3 doses with the interval of 14 days	0.654(0.260)**	0.291**	0.012**	1.923	1.155	3.203
3 doses with the interval of 28 days	0.386(0.202)*	0.172*	0.056*	1.471	0.991	2.185
Effectiveness (70 percent)			< 0.001***			
80%	− 0.805(0.154)***	− 0.352***	< 0.001***	0.447	0.33	0.605
90%	0.317(0.150)**	0.139**	0.034**	1.373	1.024	1.842
Constant	0.801(0.114)***		< 0.001***	2.229		

*p < 0.1.

**p < 0.05.

***p < 0.001.

^a^The levels in brackets after each variable (i.e. risk of adverse reaction, protective duration, vaccine injection time, and effectiveness) in Table 2 were reference value.

### Main preference

By incorporating socio demographic characteristics (including region, self-health assessment, and the number of vaccinated household members) into the model using a stepwise approach, we constructed a new model featuring interaction terms. The regression results are shown in Table 3, in which most variables were significantly associated with vaccination preference except for the risk of adverse reaction. Among the socio demographic variables, respondents' willingness to vaccinate decreased as the number of vaccinated household members increased, whilst the urban residents were less willing to be vaccinated than the rural residents. In this study, the level of “very good” is the reference level for the variable “self-health assessment”, and it is interesting to note that people in poor health were more likely to get vaccinated. Through a comparison of the final valid interaction terms, we found that the more severe the risk of adverse reaction was, the less likely people were to be vaccinated, across both rural and urban areas. Secondly, the interaction term properties of the number of vaccinated household members and the risk of adverse reaction suggested that these two factors had complex effects on vaccination intentions. Thirdly, a decrease in the level of health status enhanced the positive influence of protective duration on vaccination preference. Overall, the inclusion of socio demographic variables influenced how the attributes of the vaccine affect people's vaccination preferences (even from a negative to a positive effect). Additionally, considering the representativeness of the sample, we also divided different subgroups according to attributes such as gender and age group and did robustness tests, and the results were consistent with the results of the full sample.

**Table 3:** Regression results of DCE.

	B	P value	Exp (B)	95% CI for Exp (B)	
				Lower	Upper
Region	− 1.110	0.006**	0.330	0.149	0.729
Number of vaccinated household members		< 0.001***			
1 vaccinated member	− 0.528	0.352	0.590	0.194	1.792
2 vaccinated members	− 1.009	0.092*	0.365	0.113	1.177
3 vaccinated members	− 1.651	< 0.001***	0.192	0.100	0.366
Risk of adverse reaction		0.276			
Local or systemic adverse reactions	0.104	0.913	1.110	0.171	7.191
Severe adverse reactions	− 0.871	0.126	0.418	0.137	1.277
Injection time		0.013*			
2 doses with the interval of 14/28 days	1.302	0.150	3.675	0.624	21.661
3 doses with the interval of 14 days	1.465	0.106	4.329	0.733	25.566
3 doses with the interval of 28 days	0.429	0.038**	1.535	1.023	2.302
Effectiveness		< 0.001***			
80% percent	− 0.818	< 0.001***	0.441	0.324	0.601
90% percent	0.322	0.036**	1.380	1.022	1.864
95% percent	− 0.777	0.394	0.460	0.077	2.743
Region*Adverse reaction		< 0.001***			
Urban region*local or systemic adverse reactions	− 0.969	0.025**	0.380	0.162	0.887
Urban region*severe adverse reactions	− 2.929	< 0.001***	0.053	0.017	0.165
Number of vaccinated household members*Adverse reaction		< 0.001***			
1 vaccinated member*local or systemic adverse reactions	− 2.527	< 0.001***	0.080	0.037	0.171
1 vaccinated member*severe adverse reactions	− 0.460	0.419	0.631	0.207	1.925
2 vaccinated member*local or systemic adverse reactions	0.263	0.641	1.300	0.431	3.928
2 vaccinated member*severe adverse reactions	− 1.900	< 0.001***	0.150	0.055	0.409
3 vaccinated member*local or systemic adverse reactions	− 0.133	0.827	0.875	0.265	2.888
3 vaccinated member*severe adverse reactions	− 0.977	0.079*	0.377	0.127	1.119
Protective duration		0.004**			
18 months	3.298	0.004**	27.063	2.852	256.823
Self-health assessment		0.014**			
Good	− 1.461	0.109	0.232	0.039	1.385
Fair	− 0.945	0.299	0.389	0.065	2.315
Poor	− 0.278	0.784	0.757	0.103	5.546
Very poor	− 0.353	0.218	0.702	0.400	1.232
Self-health assessment*Protective duration		< 0.001***			
Good*18 months	− 2.440	0.038**	0.087	0.009	0.877
Good*24 months	− 1.684	0.074*	0.186	0.029	1.180
Fair*18 months	1.468	0.106	4.340	0.732	25.715
Fair*24 months	− 1.227	0.383	0.293	0.019	4.609
Poor*18 months	0.820	0.365	2.271	0.385	13.410
Poor*24 months	− 1.213	0.387	0.297	0.019	4.650
Very poor*18 months	0.017	0.987	1.017	0.138	7.509
Very poor*24 months	− 1.096	0.477	0.334	0.016	6.829
Constant	2.253	< 0.001***	9.512		

*p < 0.1.

**p < 0.05.

***p < 0.001.

In summary, the above results suggest that people weighed both the attributes of the vaccine and socio demographic factors when deciding whether to get vaccinated, and also that different combinations of variables had different effects on the vaccination preferences of the population. For the elderly people, the safety of the vaccine, the number of vaccinated household members, and their self-health assessment had a greater impact on vaccination intentions and preferences.

## Discussion

This study reports the results of a DCE study quantifying the elderly respondents’ stated preference for the COVID-19 vaccination program. To our knowledge, this is the first study to investigate the elderly population's preferences for selecting such vaccination programs using DCE. The study results showed that the respondents’ vaccination probability increased with an increase in the vaccine's protective duration and with a decrease in the risk of adverse reaction. The model of interactive variables further suggests that the region, the number of family members’ vaccinations and self-health assessment influence elderly people’s preferences for COVID-19 vaccination.

### Findings in the model of vaccine attributes

In the model of vaccine attributes, an adverse reaction is the most important vaccine attribute that affects participants’ preferences for the COVID-19 vaccine in China. The coefficient of adverse reactions is negative, indicating that when adverse reactions to the vaccine change from no side effects to local or systemic adverse reactions, and from no side effects to severe adverse reactions, peoples’ willingness to undergo vaccination will decrease. Among them, the coefficient of serious adverse reaction is greater than the absolute value of the coefficient of slight or systemic adverse reaction, indicating the individuals in the study sample are more reluctant to vaccinate new crown vaccine when severe immune reaction may occur than local adverse reaction and systemic side reaction. As there are other reported adverse reactions to the COVID-19 vaccine, elderly people may pay closer attention to this attribute, a result that is consistent with previous studies^19–21^. Whilst the research subjects in other studies are mainly young and middle-aged people, our research focuses on the elderly population and will be complementary to the existing literature. This result shows that more serious adverse reactions tend to reduce the population's willingness to receive the COVID-19 vaccine.

It can be seen that the correlation between vaccination willingness and vaccine injection times is both significant and positive, thus indicating that compared with one injection, the elderly are more likely to choose to be injected two or more times. In addition, the results show that people prefer a vaccination course split into three doses with an interval of 14 days between, as opposed to two doses with an interval of 14 or 28 days. This result is inconsistent with the existing studies on the public’s preference for COVID-19 vaccines^17,20,22^. One recent piece of research also found that people are more willing to undergo vaccination with fewer injection times^23^. According to the health belief model, in this study, the opposite results may stem from the impact of news reports on people's cognition of vaccination^8^. It should also be noted that the adverse reactions caused by one injection are stronger and that vaccines with multiple doses and long interval times may have provided better immunity than those with one injection time. The selected elderly group will link the doses and interval time with the existing reports, leading them to select the vaccine with more injection times. Furthermore, protective duration has a positive effect on elderly people’s willingness to undergo vaccination, which is consistent with the previous studies^24–26^.

The results of the vaccine attributes model indicate that adverse reactions, doses, and protective duration are correlated with elderly people’s preferences for the COVID-19 vaccine. Considering that the elderly population are vulnerable to COVID-19 and much of this group in China remains unvaccinated, this study offers suggestions pertaining to the levels of vaccine attributes for policymakers to involve more elderly people in vaccination in the future and improve the COVID-19 vaccine uptake rate. Additionally, healthcare practitioners could emphasize the specific attributes of COVID-19 vaccines, such as adverse effects, to increase the acceptance of the public and uptake rate.

### Findings in the model of interactive variables

The model of interactive variables shows that region is the most important demographic attribute affecting participants’ preference for the COVID-19 vaccine in China. To be precise, those people living in rural areas exhibited a greater preference for inoculation over their urban counterparts, which is inconsistent with the previous studies^20,27^. More convenient medical service facilities and richer information about medical services in urban areas lead to differences in residents' health perceptions and health-related behaviours^28^. Elderly residents in urban may have access to more information related to vaccines and epidemics, and still harboured greater uncertainty and mistrust about vaccine safety. At the same time, older people in urban areas had a higher level of health care coverage than rural areas, and could access a higher level of medical assistance in the emergency, and were therefore less likely to be vaccinated. In addition, it can be seen that the number of family members’ vaccinations is negatively correlated with people’s vaccination willingness, a finding that is consistent with another survey conducted in China^29^. According to the health decision-making model^30^, the health of family members will influence the perception of the threat of COVID-19 infections in elderly people. And in the health belief model, the likelihood of people adopting health behaviours is related to perceived susceptibility. When more household members are vaccinated against COVID-19, the less likely they are to be infected. So elderly people could feel more protected and believe they have a lower likelihood of getting the disease (i.e. the lower risk perception). Alternatively, this may indicate that elderly people tend to “free-ride” on the protection by others around them as a strategic response to infectious disease threats. However, another study found that having at least one vaccinated non-elderly family member was strongly associated with the vaccination of the elderly person in the family^31^, which is in contrast with the trend identified in the present study. This suggests that the influence of family members on people's willingness to be vaccinated deserves further detailed exploration.

An effective self-health assessment will have a significant negative impact on people’s vaccination willingness: the worse the self-health assessment, the more likely people are to be vaccinated. This finding is consistent with those put forward in a previous study^25^. Another study also concluded that people with health problems (who are likely to have a lower self-health assessment) tend to get a vaccine for seasonal influenza^32^. Older people in good health may have lower perceived susceptibility and perceived severity, that is to say, they perceive their likelihood of infection and the severity of the consequences of infection to be low. However, people in poor health may be more willing to be vaccinated because they fear that they are more susceptible to infection due to their low immune system or other reasons.

The interaction between region and adverse reaction is a significant indicator that the people living in towns and in urban areas have different senses of utility in terms of adverse reactions. Under the same known protective duration, people with poor health status are more willing to be vaccinated than people in good health. In addition, there is the interaction term between the number of family members’ vaccinations and the adverse reactions, indicating that under the same adverse reaction level of local or systemic, the fewer people that are vaccinated in the family, the more reluctant the elderly are to get a COVID-19 vaccination. This result may be due to the fact that when more family members are vaccinated, elderly people adopt the mentality that adverse reactions are not as severe as the media suggests, indicating that they will view vaccinations as less harmful. Therefore, according to the health decision-making model^30^, the elderly may increase their willingness to vaccinate.

The results of this model provide information about the effect of demographic variables on elderly people’s COVID-19 vaccination preferences. Going forward, governments are able to take certain groups of people into consideration and create more specific policies. Using these interactive results, it is also possible for governments to adopt different vaccination strategies for different groups of elderly people because they present the distinctive vaccine attribute preferences among elderly people in detail.

### Strengths and limitations

Although a handful studies have explored the factors affect individuals’ decisions on the COVID-19 vaccine, few of them paid adequate attention to the importance of the elderly people’s preferences about various attributes of the COVID-19 vaccine. This study found that the adverse effect of vaccines is the most influential factor affecting the choice of COVID-19 vaccines among the elderly Chinese population, whilst protective duration and vaccine injection times were not significant. This study adopted the Discrete Choice Experiment (DCE) method, which is widely used in patient-oriented research to estimate the preferences of complex objects by building assumptive scenarios. Most of the DCE studies carried out in China have focused on the use of health services, the choice of workplace, and the establishment of the healthcare model, with very few investigating decision-making. Therefore, our efforts will provide more information on COVID-19 vaccination decision-making for elderly people in China.

Despite the advances made here, the limitations of the present study must also be addressed. First, due to methodological deficiency inherent in the DCE, the findings of this study only represent participants’ self-reported willingness to take COVID-19 vaccines. It may be the case that the subjects’ actual behaviors in real life may significantly differ from their stated preferences. With this in mind, it would be interesting for further research to compare the differences between participants’ stated preferences and their actual behaviors toward COVID-19 vaccination and use a more heterogeneous analysis approach. Second, the target population was the elderly and the majority of our respondents are from urban areas, such that there is insufficient data on the elderly in rural China, which implies that the results of this survey may not cover the entire population and the generalizability could be reduced. Finally, a sample from only one community suggests may limit the generalizability of the findings. Further research could explore whether our findings could be extended to populations with different health statuses or in different geographical locations.

## Methods

### Study population

The target population of this study is middle-aged and elderly people aged 50 years and above, since there was increased risk of severe illness from COVID-19 among the elderly population, according to the evidence from China and the US^33,34^. The DCE survey was conducted in Haidian District, Beijing from January 24 to March 10, 2021, with a random sample of middle-aged and older adults aged 50 and above in the community of Renmin University of China. Considering the results from the pretest, the attributes and levels included, we assume a sample of 250 respondents will be sufficient to achieve the study’s aims. As a results, it involved 298 participants. To ensure the accuracy of the survey, we excluded any surveys with incomplete or contradictory answers and those who could not read or understand the whole questionnaire questions. Ultimately, 293 valid questionnaires were obtained. Since vaccine hesitancy is described as a continuum between complete acceptance and refusal, the study covered those unintending to get vaccinated. To recruit samples, we used a purposeful sampling to ensure highest variability in the initial data. We compared the demographic characteristics of our samples and the statistics from the Chinese Health Year Book 2021^35^, and found the included the main demographic features of our samples were comparable with the general elderly population in urban areas of China, specifically with the proportion of the population aged 65 and over in Beijing, and with the educational level of urban residents.

### Discrete choice experiment

A Discrete Choice Experiment (DCE) is a form of survey that has been widely used in patient-oriented research^20,25^. The present study designed a DCE tool with five attributes (the risk of adverse effects, protective duration, injection doses, injection period, and effectiveness) and corresponding levels (the number of injection doses is divided into three levels, as 1, 2, 3 doses) according to relevant COVID-19 vaccine research and practice^24^. Individuals’ preferences were assumed to be determined by different attributes and levels^36,37^. In a DCE, respondents are asked to make a series of trade-offs between two realistic or hypothetical vaccine options. The results derived from the research then enable the researchers to determine the relative significance of the included levels and attributes^38^. In this study, there were 12 sets of trade-offs by eliminating incompatible vaccine combinations, of which the following was one set of specific scenarios: Case 1: The vaccine had no side effects, had a protective duration of 6 months, was given in 1 dose and the effectiveness rate was 70%. Case 2: The vaccine had systemic adverse reactions, the protective duration was 12 months, 2 doses were given at an interval of 28 days, and the effectiveness rate was 80%.

### Attributes and levels

In this study, the attributes were selected and their levels were set based on a literature review related to vaccination intention^20,21,25,39^, interviews with experts in relevant fields, and current COVID-19 vaccine development progress and interviews with experts in relevant fields. In the initial phase of this survey, the world was witnessing a growing variety of COVID-19 vaccine developments, but most were in pre-clinical stages. We constructed a basis for the classification by comparing and summarising the main levels of properties of different vaccines according to the statistics of WHO and several literature. Thereafter, expert opinion on vaccine property and level settings was used to finalise the attributes and levels used in this study. It should also be noted that we excluded the attribute of price given that the context of this study was in China and that COVID-19 vaccines are provided to Chinese residents free of charge. The final set of five attributes with three to four levels for each is presented in Table 4. By referring to the extensive body of medical-related literature, we have classified the adverse effects into three degrees (no effects, local or systemic adverse reactions, and severe adverse reactions) and the protective duration into four levels (6 or 12–18–24 months)^20,40–42^. The injection period pertains to the interval between two vaccination doses and the effectiveness means the level of reduction in the risk of developing symptoms of infection in vaccinated individuals in a clinical or real-life setting. Based on current vaccine development standards and clinical data, we set the bottom level of effectiveness as 70%^43,44^.

**Table 4:** Attributes and Levels of the DCE study.

Attributes	Levels
Risk of adverse reaction	No side effects—Local or systemic adverse reactions (pain at the vaccination site, local muscle,or fever, headache, fatigue, muscle aches and pains, minor colds, etc.)—Severe adverse reactions (hypersensitivity reactions, autoimmune reactions, other rare severe allergic reactions, etc.)
Protective duration	6 or 12–18–24 months
Injection doses	1–2–3 doses
Injection period	0–14–28 days
Effectiveness	70%–80%–90%–95%

### DCE experiment design

Determining attributes and levels is the first step when conducting a Discrete Choice Experiment. The second step is to generate the DCE option set by carrying out the experiment with the identified attributes and levels using the relevant software. The DCE design in this study contained five attributes and 3–4 levels each. A full-factorial design using all the attributes and levels results in 576 (4 × 4 × 3 × 3 × 4) possible profiles and provides 165,600 (576 × 575 × 1/2) pairwise choice sets for selection. Using Allpairs software, 22 pairwise choice sets were constructed following an orthogonal test. After removing the unrealistic combinations of several attributes and levels (e.g. 2 injection doses but the injection interval is 0 days), 13 vaccine combinations were obtained. Of these, one option with a relatively average level of each attribute was selected as a control, whilst the other 12 combinations were paired with it to form 12 pairs of trade-offs.

### Questionnaire

In order to improve the reliability and validity of our questionnaire, we first reviewed the existing literature on this area^45,46^. Based on the suggestions and discussion presented in these studies, we conducted a pilot study to ascertain whether the attributes were appropriate and also whether the questionnaire was understandable and acceptable. The formal questionnaire consisted of three parts: (1) demographic characteristics of the respondent; (2) health-related questions; (3) the DCE. Moreover, demographic characteristics included gender, age, income, region, educational level, and occupation, whilst the vaccination-related statements contained the history of chronic disease, health condition, medical insurance, fixed places for medical treatment, and the vaccination condition of family members. These patient characteristics were all hypothesized to have an impact on vaccination uptake, and as such, were employed as variables in the data analysis. The final section of the questionnaire required respondents to make a series of DCE choices in a hypothetical vaccination scenario, an example was provided in Supplementary Table S1. Before all the choice tasks, the question “Are you willing to get the COVID-19 vaccination?” was provided to allow respondents to “opt out” because, in real life, respondents are not obliged to undergo vaccination against COVID-19. If the respondents were willing to receive a vaccination, they were then asked to consider all alternatives and choose their preferred COVID-19 vaccine combination from each pair.

### Statistical analysis

This study utilized a multinomial logistic regression model to analyze the data. First, we performed a basic logistic regression analysis, using all the attributes and levels included in the present study to explore the direction and degree of influence of separate attributes and levels on the elderly study population’s vaccination preferences. Secondly, several variables relating to background characteristics and vaccine-related attributes including region, the number of vaccinated household members and self-health assessment were incorporated to form a series of interaction items. Doing so allowed us to explore the relationship between attributes and demographic characteristics as well as between attributes. For the coefficients, the statistical significance results (p-value < 0.05) indicate that the attribute has an influence on decision-making in the DCE. The value of the coefficient represents a positive or negative effect of the attribute on utility. SPSS Statistics 26 Software was used to carry out the analysis. Considering the representativeness of the sample, we also ran sensitivity analysis to check on robustness of the finding.

### Ethics statement and consent to participate

The experiment in this study was approved by Renmin University Health Policy Research and Evaluation Center (RUC HPRE IRB) and performed in accordance with the Helsinki Declaration of 1975, as revised in 2000. Informed consent was obtained from all participants included in the study. All individual participants provided informed consent to participate and use of their data for publication.

## Conclusion

Although the COVID-19 vaccination drive in China is free of charge, not all elderly people have been inoculated. Since the COVID-19 virus is still mutating, vaccination is necessary for every person, especially the elderly, as a susceptible population. This study employed a Discrete Choice Experiment (DCE) to evaluate the role of vaccine-related attributes in affecting the preferences of the Chinese aging population for COVID-19 vaccines. According to the results, the vaccination willingness was found to vary depending on both the specific attributes of the vaccine and the participants’ demographic characteristics, such as the difference of urban and rural and people’s health status. This study also underscore the importance of elderly people’s willingness and their families’ influence on the decision to vaccinate. Additionally, we found that group differences also had an impact on vaccination intentions. Therefore, the government could enhance education on vaccine knowledge and policies for older people as a way to improve scientific and comprehensive knowledge of the coronavirus and vaccine, thereby increasing vaccination rates. Meanwhile, the vaccine performance should be enhanced to reduce the adverse effects of the vaccine on human body and increase people's conviction of the safety of the vaccine. Thirdly, differences in group concerns about the vaccine attributes could also be a potential factor considered in the introduction of vaccination policies. To conclude, this study provides a possible means for policymakers to improve the COVID-19 vaccine injection rate as well as other vaccines, particularly for the older Chinese population. Future studies should further expand the scope of the survey population and incorporate new vaccine policies in order to secure a better understanding of the vaccination preferences of elderly people in China and improve vaccination intentions and inoculation rates among older citizens (Supplementary Table S1).

## Supplementary Information

Supplementary Table S1.

## Data Availability

The datasets generated and analyzed during the current study are available from the corresponding author upon reasonable request.
