# Supplementary material for: The COVID-19 vaccination decision-making preferences of elderly people: a discrete choice experiment
Source: Sci Rep. 2023 Mar 31;13:5242. doi: 10.1038/s41598-023-32471-1 (PMC10063931; doi:10.1038/s41598-023-32471-1)
Supplement: Supplementary file 1 — Supplementary Table S1. [file 41598_2023_32471_MOESM1_ESM.docx]

**Supplementary information file 1**

Title: The COVID-19 vaccination decision-making preferences of elderly people: a discrete choice experiment

Author List: Yuhan Chen^1^, Jimeng Wang^1^, Meixi Yi^1^, Hongteng Xu^1^, Hailun Liang^1,^*

^1^Renmin University of China, Beijing, 100872, China

*hliang@ruc.edu.cn

The table below is an example of a choice scenario, derived from a specific set of scenarios from the actual survey. In the actual survey, a total of 12 similar sets of choice scenarios were developed and the respondent was asked to select a combination of vaccine attributes that he or she would prefer to be vaccinated in each specific set of scenarios.

|  | Vaccine A | Vaccine B |
| --- | --- | --- |
| Risk of adverse reaction | Systemic adverse reactions (fever, headache, fatigue, muscle aches and pains, minor colds, etc.) | No side effects |
| Protective duration | 12 | 6 |
| Injection doses | 2 | 1 |
| Injection period | 28 | 0 |
| Effectiveness | 80% | 70% |
| I chose for: | □ | □ |

**Supplementary Table S1.** An example of a choice scenario.
